# Minimum epistasis interpolation for sequence-function relationships

**DOI:** 10.1038/s41467-020-15512-5

**Published:** 2020-04-14

**Authors:** Juannan Zhou, David M. McCandlish

**Affiliations:** Simons Center for Quantitative Biology, Cold Spring Harbor Laboratory, Cold Spring Harbor, NY 11724 USA

**Keywords:** Computational biology and bioinformatics, Evolutionary genetics, Evolutionary theory, Epistasis

## Abstract

Massively parallel phenotyping assays have provided unprecedented insight into how multiple mutations combine to determine biological function. While such assays can measure phenotypes for thousands to millions of genotypes in a single experiment, in practice these measurements are not exhaustive, so that there is a need for techniques to impute values for genotypes whose phenotypes have not been directly assayed. Here, we present an imputation method based on inferring the least epistatic possible sequence-function relationship compatible with the data. In particular, we infer the reconstruction where mutational effects change as little as possible across adjacent genetic backgrounds. The resulting models can capture complex higher-order genetic interactions near the data, but approach additivity where data is sparse or absent. We apply the method to high-throughput transcription factor binding assays and use it to explore a fitness landscape for protein G.

## Abstract (web summary)

High-throughput combinatorial mutagenesis assays are useful to screen the function of many different sequences but they are not exhaustive. Here, Zhou and McCandlish develop a method to impute such missing genotype-phenotype data based on inferring the least epistatic sequence-function relationship.

## Introduction

Recent advances in quantification via next-generation sequencing have allowed the proliferation of high-throughput combinatorial mutagenesis assays that measure molecular function for tens of thousands to millions of sequences simultaneously^1^. These assays have been applied to many different classes of functional elements, including protein-coding sequences^2–13^, RNAs^14–18^, and regulatory or splicing elements^19–22^. However, in practice, due to both the vastness of sequence space and the limitations of techniques for library preparation, such experiments typically result in missing measurements for a subset of possible genotypes.

Making accurate phenotypic predictions for these missing sequences is a difficult problem because the effect of any given mutation often depends on which other mutations are already present in the sequence, a phenomenon known as epistasis^23–25^. In the special case where such interactions are limited to occuring between pairs of sites, the prediction problem can be solved using regularized regression^26^—a technique that has sometimes performed quite well^27,28^. However, there is now abundant evidence that adding pairwise interaction terms to an otherwise additive model is not sufficient to capture the complex interdependencies between mutations observed in the empirical data^10,24,29–39^.

In principle, these “higher-order” interactions can be captured by adding interactions between three or more sites to standard regression models, but this leads to problems in interpretability and overfitting because the number of such terms grows rapidly with increasing interaction order^26^. Another strategy has been to assume that the observed phenotype is a simple nonlinear function of some underlying nonepistatic trait^32,40^, a pattern of epistasis known as univariate^8,24^, nonspecific^31^, or global^40,41^ epistasis, which appears to be well-suited primarily to sequence–function relationships that are essentially noised versions of single-peaked landscapes. Finally, a variety of machine-learning techniques^8,12,42–45^ have been employed that can fit more complex forms of epistasis than global epistasis or pairwise interaction models. However, these require substantial tuning and the resulting models exhibit behavior that is difficult to interpret.

Here, we present a method for fitting sequence–function relationships that includes epistatic interactions of all orders but whose predictions are nonetheless conservative, which has no tunable parameters, and which is simple enough to provide formal mathematical guarantees on its behavior. The main idea is to assign the missing phenotypic values in such a way that the effects of mutations are as consistent across mutationally adjacent genetic backgrounds as possible. We achieve this by minimizing the expected squared epistatic coefficient for random pairs of mutations over all possible genetic backgrounds, a minimization problem that comes down to solving a single set of coupled linear equations. The end result is a model that can provide a complicated fit where data are abundant, but which approaches additivity in regions of sequence space where data are sparse or absent.

In what follows, we first describe our modeling technique and its mathematical properties. We then compare our method with regression models in terms of predictive power and behavior, using a variety of simulated data sets, including a simple biophysical model for transcriptional regulation^46^ and a model featuring sparse, higher-order interactions^36^. Turning to empirical data, we first conduct an in-depth analysis of a deep mutational scanning data set from protein GB1^34^, a system known to contain a particularly complex pattern of genetic interaction. Combining our imputation and a previously proposed visualization technique^47^, we show that the complex structure of epistasis observed in this data set can be well-understood in terms of a simple qualitative model consisting of three fitness peaks where the landscape is locally additive in the vicinity of each peak. Finally, to provide a broader demonstration of the performance of our technique, we apply our method to hight-throughput DNA-binding preference assays for a set of 1121 transcription factors^48^, a collection of measurements which has become a model system for understanding genotype–phenotype relationships^49,50^. We show that minimum epistasis interpolation has better predictive power and a lower false discovery rate than low-order regression models for the vast majority of these transcription factor data sets.

## Results

### Minimum epistasis interpolation

Given phenotypic observations on a subset of genotypes, our goal is to assign phenotypic values to all unobserved genotypes in such a manner that mutational effects change as little as possible between mutationally adjacent genetic backgrounds. To understand our solution to this problem, it is helpful to think about the simplest possible case where sequence space consists of two bi-allelic loci and hence four possible genotypes. We assume we have observed phenotypes for the wild type (*f*_ab_) and both single mutants (*f*_Ab_ and *f*_aB_), and we want to predict the phenotype of the double mutant (*f*_AB_).

**Fig. 1:**
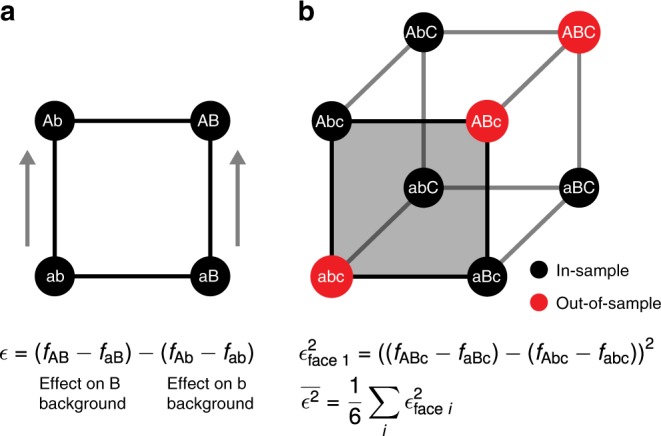
Minimizing average local epistasis. **a** The classical epistatic coefficient *ϵ* measures the difference in the effect of a mutation between two adjacent genetic backgrounds. Here *ϵ* is shown as the difference between the effect of an a → A mutation on a *B* versus *b* background. **b** Larger spaces of genotypes can be decomposed into faces consisting of a wild-type sequence, two single mutants and a double mutant; one such face is highlighted in gray. For each face, we quantify epistasis locally by calculating the corresponding value of *ϵ*^2^. We then quantify the total amount of epistasis for the sequence–function relationship by taking the average of these values across all faces, $\overline{{\epsilon }^{2}}$. By assigning phenotypic values for the out-of-sample genotypes that minimize $\overline{{\epsilon }^{2}}$, we infer the least epistatic sequence–function relationship compatible with the data in the sense that the average squared difference in the effects of mutations between adjacent genetic backgrounds is as small as possible.

For this simple case, we can measure the change in the effect of a mutation across genetic backgrounds using the traditional epistatic coefficient^51^:

1 $$\epsilon =({f}_{{\rm{AB}}}-{f}_{{\rm{aB}}})-({f}_{{\rm{Ab}}}-{f}_{{\rm{ab}}}),$$

which is just the change in the effect of an a → A mutation between the *b* and *B* backgrounds (Fig. 1a), and is also equal to the change in the effect of a b → B mutation between the *a* and *A* backgrounds. However, the sign of *ϵ* depends on which sequence we have chosen as the wild type, so if we want a reference-free measure of how much mutational effects change with genetic background we can instead use the squared quantity *ϵ*^2^, which is also proportional to the mean-square error of a nonepistatic model fit to these four genotypes.

Since we are trying to predict the phenotype for AB by minimizing the change in the effects of each mutation across genetic backgrounds, we can do so by choosing the prediction that minimizes *ϵ*^2^. In fact, for this simple case minimizing *ϵ*^2^ yields *ϵ* = 0. This results in the classical additive prediction ${\widehat{f}}_{{\rm{AB}}}$:

2 $${\widehat{f}}_{{\rm{AB}}}={f}_{{\rm{ab}}}+({f}_{{\rm{Ab}}}-{f}_{{\rm{ab}}})+({f}_{{\rm{aB}}}-{f}_{{\rm{ab}}})$$

where, in particular, the predicted double-mutant phenotype is equal to the observed wild-type phenotype plus the sum of the observed single-mutant effects.

How can we generalize this classical additive prediction for the two-locus bi-allelic case to larger sequence spaces and more complex patterns of missing data? Unless our observations are themselves drawn from an additive model, in this more general context it will typically be impossible to find a set of predictions for the missing genotypes such that *ϵ* = 0 for all pairs of mutations on all genetic backgrounds. However, even if the effects of mutations are not absolutely consistent across genetic backgrounds, we can still look for a set of predictions where the effects of mutations are as consistent as possible.

In particular, for each mutation and pair of adjacent genetic backgrounds, we can define a corresponding value of *ϵ*^2^ that measures the squared difference in the effect of that mutation between the two backgrounds. We then search for the set of predictions that minimizes the expected value of this squared epistatic coefficient across all possible combinations of mutations and pairs of backgrounds. That is, we are looking for a set of predictions that make the reconstructed sequence–function relationship as locally additive as our observations allow, without imposing global additivity or any specific assumptions about the form of epistasis.

More precisely, for genotypes with *l* sites and *α* possible alleles at each site, we can consider the space of possible sequences as a (generalized) hypercube or Hamming graph with $$s=\left(\begin{array}{l}l\\ 2\end{array}\right){\left(\begin{array}{l}\alpha \\ 2\end{array}\right)}^{2}{\alpha }^{l-2}$$ “faces”, each of which consists of four genotypes that can be described as a wild-type sequence together with two single mutants and a double mutant, Fig. 1b. Any vector **f** that assigns phenotypes to the *α*^*l*^ possible genotypes also defines a value of *ϵ*^2^ for each of these faces and we denote the average value of *ϵ*^2^ over all such faces as $\overline{{\epsilon }^{2}}({\bf{f}})$, a quantity which is also equal to the average squared change in the effect of a mutation between two adjacent genetic backgrounds.

Thus, to generalize our solution for the two-locus bi-allelic case with one missing genotype to larger sequence spaces and arbitrary geometric arrangements of the missing data, we want to find the value of **f** that matches our observed phenotypes where available, but otherwise minimizes $\overline{{\epsilon }^{2}}({\bf{f}})$. To do this, we note that $\overline{{\epsilon }^{2}}({\bf{f}})$ is non-negative, since the *ϵ*^2^ for each face is non-negative, and that the formula for the *ϵ*^2^ of each face is a second-degree polynomial and thus so is $\overline{{\epsilon }^{2}}({\bf{f}})$. As a result, our constrained minimization problem is in fact a positive semi-definite quadratic minimization problem with an equality constraint, a form of problem that has an analytical solution^52^ based on solving a single set of coupled linear equations (see “Methods”).

In particular, if we write the set of known genotypes as *B* and the minimum epistasis interpolation solution as $\widehat{{\bf{f}}}$, we first assign $\widehat{{\bf{f}}}(i)={\bf{f}}(i)$ for *i* ∈ *B* to satisfy the constraint that our solution is equal to the observed phenotypic value when available. Then, for each *i* ∉ *B*, the minimum epistasis reconstruction of the sequence–function relationship is given by setting $\widehat{{\bf{f}}}$ to the solution of the following *α*^*l*^ − ∣*B*∣ equations (one equation for each *i* ∉ *B*, see “Methods”):

3 $$\sum_{j\notin B}\ c(i,j)\ \widehat{{\bf{f}}}(j)=-\sum_{j\in B}\ c(i,j)\ {\bf{f}}(j),$$

where the values of *c*(*i*, *j*) depend only on the Hamming distance between genotypes *i* and *j* and are given by:

4 $$c(i,j)=\left\{\begin{array}{ll}l(l-1){(\alpha -1)}^{2}/2&i=j\\ -(l-1)(\alpha -1)&i\;{\rm{is}}\ {\rm{at}}\ {\rm{Hamming}}\ {\rm{distance}}\ 1\ {\rm{from}}\;j\\ 1&i\;{\rm{is}}\ {\rm{at}}\ {\rm{Hamming}}\ {\rm{distance}}\ 2\;{\rm{from}}\ j\\ 0&{\rm{otherwise}}.\end{array}\right.$$

The above calculation comes down to solving a set of *α*^*l*^ − ∣*B*∣ linear equations, and hence scales cubically with the number of unobserved genotypes, *α*^*l*^ − ∣*B*∣. While this approach can readily be applied to moderately sized sequence spaces (e.g. *α*^*l*^ less than a million), the exponential dependence of the number of possible genotypes on the sequence length *l* makes this straight-forward approach impractical for longer sequences. Nonetheless, we can show that the minimization problem can in fact be kernelized to remove this exponential dependence on *l*, so that ultimately the computational complexity scales linearly in *l* and cubically in the number of observed genotypes ∣*B*∣ (see Supplementary Methods Proposition 2). Moreover, these equations have a unique solution if and only if the least squares fit of the corresponding nonepistatic model has a unique solution (see Supplementary Methods Proposition 1).

### Properties of the interpolation solution

Because of its mathematical simplicity, we can in fact provide several guarantees for the properties of this minimum epistasis interpolation solution.

First, consider some focal genotype *i*. This genotype is a member of $$\left(\begin{array}{l}l\\ 2\end{array}\right){(\alpha -1)}^{2}$$ faces, and the phenotypes of the three other genotypes in each face can be used to derive a nonepistatic prediction for the phenotype at *i*. Since these predictions are not necessarily all the same, we can take their mean to produce the average local nonepistatic prediction for genotype *i*. Perhaps surprisingly, the solution to our constrained minimization problem $\widehat{{\bf{f}}}$ has the property that for any missing genotype *i*, $\widehat{{\bf{f}}}(i)$ is exactly equal to this average local nonepistatic prediction.

Second, the above result can be reinterpreted in geometric terms based on the mean phenotype among genotypes at distance *d* from the focal genotype *i*. Letting **d**_*k*_(*i*) denote the mean value of $\widehat{{\bf{f}}}$ for sequences at distance *k* to *i*, we have

5 $$\widehat{{\bf{f}}}(i)={{\bf{d}}}_{1}(i)+({{\bf{d}}}_{1}(i)-{{\bf{d}}}_{2}(i)),$$

This is similar to a Taylor approximation around *i*, where we correct the nearest-neighbor estimate $\widehat{{\bf{f}}}(i)\approx {{\bf{d}}}_{1}(i)$ by the difference **d**_1_(*i*) − **d**_2_(*i*), which captures the average effects of the mutations carried by *i* when introduced on mutationally adjacent genetic backgrounds.

Third, the solution has an illuminating connection with the discretized heat equation and the (second-order) Laplace operator. In particular, if **L** is the discrete analog to the continuous Laplace operator (i.e. **L** is the graph Laplacian for our Hamming graph), then our constrained minimization problem is equivalent to a boundary-value problem for the second-order discrete Laplace operator **L**^2^ − *α***L** (see “Methods”). Interestingly, the solutions to boundary-value problems in continuous space for the squared Laplace operator (i.e. the biharmonic equation) are given by the thin-plate splines^53^, which are widely used in geometric morphometrics^54^ and in modeling fitness surfaces for continuous phenotypic traits^55,56^. We can understand this connection more intuitively by recognizing that the thin-plate splines can also be defined as solutions to a constrained minimization problem, in that they are the surfaces that go through a prescribed set of points while minimizing the total curvature, where total curvature is defined as the sum of squared second-order partial derivatives integrated over the entirety of the surface. Looking back at Eq. (1), we see that the classical epistatic coefficient can be interpreted as a discrete version of a second-order mixed partial derivative since it quantifies locally how much one mutation changes the effect of another. Similarly, the squared value of the classical epistatic coefficient is a measure of the local curvature of the sequence–function relationship. Because minimum epistasis interpolation is derived by minimizing the total (i.e. integrated) value of this local curvature, we see that minimum epistasis interpolation can be interpreted as a discrete analog of thin-plate splines adapted for use in sequence space.

Fourth, while our interpolation procedure on its own leaves the observed phenotypes unaltered, it is often useful to apply some sort of smoothing to the observed data, with the idea of filtering out experimental noise and simplifying the sequence–function relationship to reveal its major features. Our above observations in fact suggest a natural smoothing operator, **M** (see “Methods”), where applying **M** to a function **f** replaces the value of every sequence with its average local nonepistatic prediction. The key feature of this particular smoothing operator is that applying **M** to $\widehat{{\bf{f}}}$ leaves our out-of-sample predictions unchanged. Thus, we can choose to apply **M** to $\widehat{{\bf{f}}}$, if we prefer to smooth the in-sample data, or work directly with $\widehat{{\bf{f}}}$ to preserve fine-scale genetic interactions (e.g., any fully random component or house-of-cards component^30,40,57^, which would be largely removed by the action of the smoother). It is also possible to define our interpolation solution in terms of iteratively applying the smoother **M** to an arbitrary initial function, where after each application of the smoother the in-sample genotypes are returned to their observed values, and the procedure is continued until convergence (See Supplementary Methods Proposition 3). This provides another useful view on the interpolation procedure as the fixed point of a dynamical procedure that removes epistasis at each step.

Finally, there is the important issue of the influence of experimental noise on the interpolation method’s out-of-sample predictions. Because the interpolation predictions can be expressed as linear combinations of the measured values (see “Methods” Eq. (9)), we can derive explicit expressions for the noise-induced uncertainty in the interpolated predictions (“Methods” Eq. (10)). While sufficient for computationally quantifying the uncertainty in individual predictions, these expressions are somewhat unwieldy and depend on the particular pattern of missing data. However, to get a crude intuitive estimate of the extent of noise reduction, we can consider Eq. (5), which expresses the interpolation solution as a function of the average values at distance 1 and 2. Assuming we are in the data-dense regime so that all these values are available, and replacing the standard error of **d**_2_(*i*) by the generally larger standard error of **d**_1_(*i*), Eq. (5) gives an estimate of an *l*(*α* − 1)/5-fold reduction in prediction variance relative to experimental noise, which suggests strong noise reduction for e.g., DNA sequences of length 3 or more. In what follows we will pay special attention to the influence of noise, and show that in practice the noise-induced uncertainty in our predictions is typically substantially smaller than the measurement noise.

### Validation on simulated sequence–function relationships

**Fig. 2:**
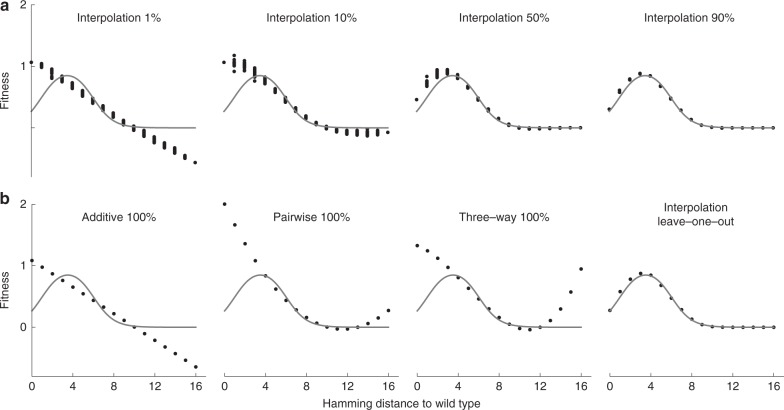
Minimum epistasis interpolation but not low-order regression models can learn the crater model for transcriptional regulation. The crater model produces a fitness landscape where fitness depends only on the Hamming distance to the wild-type sequence, with an optimum at an intermediate Hamming distance (*l* = 16 and *α* = 2; see “Methods” for other parameters). Gray curve shows the true fitness landscape. **a** Out-of-sample predictions of minimum epistasis interpolation with random subsets of 1%, 10%, 50%, and 90% of genotypes used for training. The predictions adapt to the shape of the crater landscape with increasing data density. For each distance class, at least one genotype was assigned to the test set to ensure an informative visualization of model fits. **b** Reconstruction of the crater landscape by the additive, pairwise, and three-way regression models fitted using ordinary least squares with 100% of the data. The interpolation panel shows leave-one-out results (equivalent to applying the smoother **M** to the full landscape).

To provide a simple demonstration of our interpolation technique, we first apply our method to simulated data from models of the sequence–function relationship where the phenotypic value assigned to a genotype depends only on its distance from some focal sequence. Such models are a subset of univariate^24^ or global epistasis models^40^, in that they are formed as a nonlinear transformation of an underlying additive trait (in this case, the distance from the focal sequence). However, for our purposes the most important feature of these models is that they produce a complex pattern of epistasis that can nonetheless be displayed graphically in one dimension (Fig. 2), which is helpful in getting an intuitive feeling for the behavior and characteristics of our interpolation procedure.

We begin by analyzing a biophysically inspired model for transcriptional regulation known as the crater landscape^46,58^. The model treats a single transcription factor binding site, where the fitness of the binding site is a function of the number of mismatches from the best binding sequence, and where the fitness maximum is achieved at an intermediate distance from the best binding sequence due to selection against spurious binding when the transcription factor is at a low concentration^46^ (see “Methods”). We first consider the out-of-sample behavior of our interpolation procedure as a function of the fraction of genotypes whose phenotypes are known (Fig. 2a). We see that the complexity of the model changes adaptively with the sampling density, producing essentially additive predictions when given the phenotypes for a random 1% of genotypes, but providing an increasingly close fit as the amount of training data increases.

Next, we compare our method to three commonly used regression models, namely the additive model, which assumes independent contribution of sites to fitness, and pairwise and three-way interaction models. The regression models were fit using ordinary least squares with 100% of the data in order to examine the best possible reconstruction of the true landscape given their respective model complexities. Because our interpolation procedure leaves the observed data points unchanged, as a fair comparison we make leave-one-out predictions for genotypes of each distance class by giving our method all but one genotype as training data, which is equivalent to smoothing the complete landscape using our smoother **M**. We find that while the interpolation model can provide a very good fit to this landscape, these lower-order regression models are incapable of producing a qualitatively correct approximation of the landscape, even when given 100% of the landscape as data. This occurs because the crater landscape contains interactions of all orders, and thus cannot be captured by these lower-order interaction models.

To examine the robustness of these results, we repeated the above procedure for several different global epistasis models including a Hamming ball model where the phenotype has a constant high value out to a threshold distance and then drops to a low value (Supplementary Fig. 1a), a model where the phenotype is a quadratic function of distance to the focal sequence (a simple version of Fisher’s geometric mode^59^, Supplementary Fig. 1b), and a particularly complex model where the phenotype is a sinusoidal function of distance (Supplementary Fig. 1c). In all cases at sufficiently dense sampling, the interpolation method is able to capture the basic form of the non-linearity, however, the method does exhibit some artifacts for the Hamming ball model for genotypes near the threshold distance. This occurs because the effects of mutations change suddenly at the threshold distance, violating our assumption that mutations have similar effects in nearby genetic backgrounds. In Supplementary Fig. 2, we also examine the behavior of our model on the crater landscape for longer sequences, up to length 100. We see that as we increase sequence length while fixing the number of observed sequences in each distance class, the interpolation result becomes increasingly additive, particularly at large distances from the focal sequence where the local density of observed genotypes becomes extremely small as sequence length increases. This is consistent with our general intuition that the interpolation method will behave epistatically in regions of sequence space where data are plentiful but nearly additively in regions where data are sparse or absent.

Besides having sampling rates that vary with distance from the wild-type sequence, experimental data will often exhibit substantial measurement noise. To examine our method’s behavior in a more realistic setting, we also reconsidered our analysis of the crater landscape using the data from a simulated mutagenesis experiment that includes simulated experimental noise (Supplementary Fig. 3; to appropriately treat this experimental noise in the standard regression models, we used *L*_2_ regularization where the regularization parameter was set based on tenfold cross-validation^26^). Here, we find that the interpolation method again captures the qualitative behavior of the model in the best-sampled regions of sequence space (near the focal sequence) while extrapolating additively in poorly sampled regions, and that while the variability in the predictions for any given distance class is greater than for the noise-free case (Fig. 2), it is still less than the magnitude of the experimental noise (see Supplementary Fig. 3 caption).

**Fig. 3:**
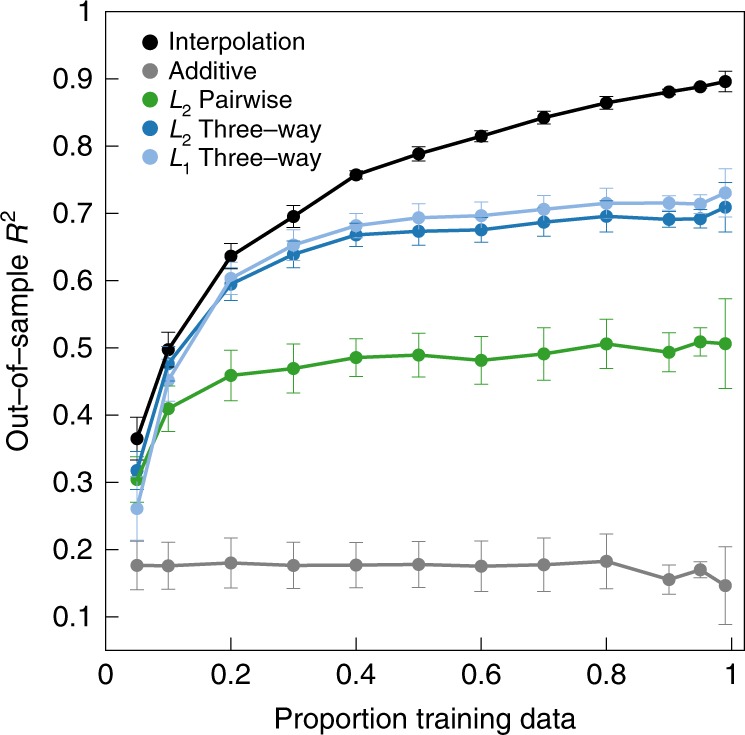
Model performance for the simulated sparse random interaction landscape with all orders of epistasis (*l* = 7, *α* = 4). *L*_2_-regularized pairwise and three-way regression models and *L*_1_-regularized three-way model were fit with regularization parameters chosen by 10-fold cross-validation. Predictive power (out-of-sample *R*^2^) is plotted as a function of the proportion of in-sample genotypes assigned as the training data. Error bars indicate one standard error around the mean, *n* = 3.

So far we have applied our interpolation method to models of global epistasis so as to allow an intuitive graphical evaluation of the performance and behavior of the method. However, these global epistasis models are a type of nonspecific epistasis that typically results in dense interactions between all mutations rather than sparse interactions between specific sets of mutations. In order to address interpolation performance for modeling-specific epistasis, we applied our method to simulated data from a sparse interaction model^36^. In this model, each possible set of alleles at each possible subset of positions can make an additive contribution to the phenotype, but almost all (90%) of the coefficients determining these contributions are set to zero, resulting in sparse interactions (see “Methods”). For comparison with the interpolation method, we fit *L*_1_-regularized three-way regression^60^ to exploit this sparse interaction structure in addition to the *L*_2_-regularized two-way and three-way regression models we fit previously. We evaluated performance by calculating the *R*^2^ for model predictions on held-out test data as each of the methods is given a larger and larger fraction of the simulated data set for training (Fig. 3). We see that at low data density the three-way *L*_1_-regularized regression model performs equally well as the interpolation model, but that at high data density the interpolation model has the best performance. Intuitively, this occurs because at low data density, the three-way *L*_1_-regularized regression can exploit the true sparse structure of the interactions whereas at high data density the interpolation model can capture higher-order interactions that the lower-order regression models cannot accommodate.

To summarize these simulation studies, the interpolation method appears to provide a highly flexible modeling framework that, given sufficient data, can capture complex patterns of both specific and nonspecific epistasis. While the model gives the most interesting results in regions of sequence space where the data are plentiful, its behavior in the data-poor regime is innocuous and similar to an additive model. We observe that the model performs worst in circumstances where there are systematic deviations from the underlying assumption that mutational effects change smoothly (e.g. the global epistasis model with a strong threshold effect), but the out-of-sample predictions of the interpolation method are nonetheless relatively insensitive to experimental noise (Supplementary Fig. 3), or, equivalently, fully random epistasis. Overall, these results suggest that the interpolation method is best suited for mid-size genotype–phenotype maps that are small enough that measurements are available for a sizable fraction of all genotypes.

### Application to protein G

Having explored the behavior of our interpolation method on simulated data, we now turn to analyzing empirical data. We begin by considering a combinatorial mutagenesis study of the IgG-binding domain of streptococcal protein G (GB1)^34^, which is a model system for studying protein folding stability and binding affinity^5,34,61,62^. By sequencing a library of protein variants before and after binding to IgG–Fc beads, this experiment^34^ attempted to assay all possible combinations of mutations at four sites (V39, D40, G41, and V54; 20^4^ = 160,000 protein variants) that had previously been shown to harbor a particularly strong and complex pattern of genetic interactions^5^. Binding scores were determined as log enrichment ratios (logarithm of ratio of counts before and after selection, normalized by subtracting the log ratio of the wild type), however, the original authors could not report binding scores for 6.6% of variants due to low coverage in the input library (ten or fewer input reads^34^).

Here, we use this data set to both predict the phenotypes for these missing sequences and to assess the performance of our method by making predictions for randomly sampled held-out data. In addition to minimum epistasis interpolation, for comparison we also fit an additive model using ordinary least squares, *L*_2_-regularized pairwise and three-way regression^26^, and *L*_1_-regularized three-way regression^60^, with regularization parameters chosen by tenfold cross-validation (see “Methods”).

**Fig. 4:**
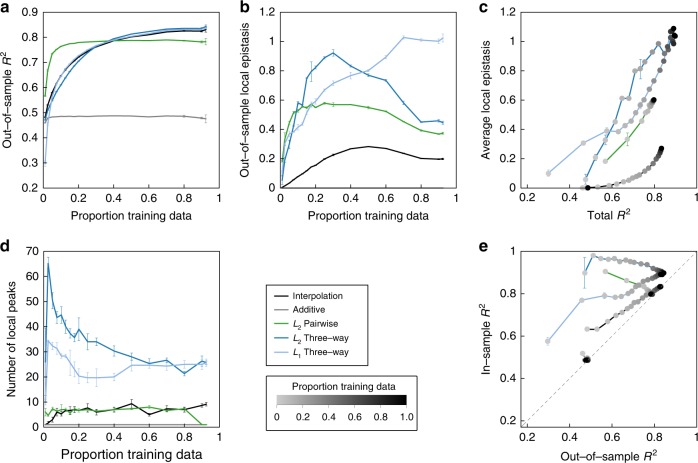
Model performance for the GB1 combinatorial mutagenesis data set. Additive models were fit using ordinary least squares. Pairwise and three-way interaction models were fit using regularized regression with regularization parameters chosen by tenfold cross-validation (see “Methods”). Points are color-coded to represent the proportion of the data randomly assigned as training. Error bars indicate one standard error around the mean. *n* = 3. **a** Predictive power (out-of-sample *R*^2^) as a function of the proportion of in-sample genotypes. **b** Mean-squared epistasis coefficients between random pairs of mutations connecting out-of-sample genotypes as a function of the proportion of in-sample genotypes. **c** Behavior across all of sequence space (both in-sample and out-of-sample) of the five models assessed using *R*^2^ between the fitted model and the complete data set (total *R*^2^) and average local epistasis ($\overline{{\epsilon }^{2}}$). Each model is represented by a curve with points corresponding to increasing proportion of the total data set assigned as training data. Note that the additive model appears at the lower left part of the plot as its total *R*^2^ quickly stabilizes and its $\overline{{\epsilon }^{2}}$ is zero by definition. **d** The number of local maxima of the reconstructed landscapes at different training data sizes. **e** Model optimism assessed by plotting in-sample *R*^2^ vs. out-of-sample *R*^2^.

We first compare the predictive power of the five models by plotting the out-of-sample *R*^2^ against training sample size, Fig. 4a. The four epistatic models substantially outperform the additive model, consistent with the high degree of epistasis previously observed for this data set. While the pairwise model produces a good fit with relatively little training data and is the best performing model when training data on less than 40% of genotypes is available, its out-of-sample *R*^2^ saturates at 0.78 and fails to improve beyond 20% training data. In contrast, the out-of-sample *R*^2^ for the three-way models and our interpolation method continue to improve and surpass the pairwise model at high data density, indicating the presence of higher-order epistasis in this data set. Overall, the predictive power of our method and the three-way models were very similar throughout the whole range of sampling, with the interpolation model having marginally better predictive power at low data density and the three-way models performing marginally better at high training data density (test-set *R*^2^ of 0.831 for interpolation, 0.843 for *L*_2_ regularized three-way regression, and 0.838 for *L*_1_ regularized regression at the largest fraction of the training data, 92.4%, with the remaining 93.4% − 92.4% = 1% of observed genotypes reserved as a test set).

However, despite the similar predictive power of the three-way and interpolation models, the interpolation achieves this predictive power using far less epistasis than the three-way models. In particular, Fig. 4b shows the mean-squared epistatic coefficients between pairs of mutations within the missing and held-out data (i.e. across all faces contained in the missing and held-out data). We see that the mean-squared epistatic coefficient for minimum epistasis interpolation is less than half of the mean squared epistatic coefficient for the three-way models across the whole range of sampling densities and that the interpolation model even has less epistasis than the pairwise model. Overall, we conclude that the predictive power of the interpolation model is quite similar to the three-way interaction models, but that the reconstruction given by the interpolation ought to be preferred because it is far smoother and hence more parsimonious.

So far we have concentrated on out-of-sample prediction, but it is sometimes also useful to consider smoothed model predictions within the data in order to reduce the effects of the experimental noise and to better reveal the large-scale features of the sequence–function relationship. While the four regression models naturally provide smoothed predictions within the sample, for our interpolation model we first predict all missing data and then apply the smoother **M** which leaves the out-of-sample predictions unchanged while replacing each in-sample observation by the average of the local nonepistatic predictions (i.e. for each genotype, we consider the nonepistatic prediction based on each possible pair of single mutations and the corresponding double mutant, and then replace its observed or inferred value with the average of these predictions).

To examine the characteristics of these smoothed landscapes across all of sequence space (both in-sample and out-of-sample), we first represented each model as a curve with points corresponding to different training data sizes, Fig. 4c, plotting both the *R*^2^ between the fitted model and the complete data set (total *R*^2^) as a measure of goodness of fit and the average squared epistasis ($\overline{{\epsilon }^{2}}$) as a measure of the ruggedness of the fitted landscapes. We see that the global behavior of the interpolation model is again quite different from that of the three-way and pairwise interaction models, and at high sampling the smoothed fit deviates from the observed data to an extent that is intermediate between these three regression models, but which is far less rugged than all of them. To provide a different view on the type of epistasis that is incorporated into these smoothed landscapes, we also considered the number of local maxima in the reconstructions (Fig. 4d). Here, our method constructed landscapes with similar number of local maxima as the pairwise model, while the three-way models produced landscapes with at least three times as many local maxima, again suggesting that our smoothed landscape is providing a simpler reconstruction than the three-way interaction models.

Finally, for the purposes of understanding the qualitative features of the sequence–function relationship, it is desirable that the characteristics of the smoothed landscape are similar across the observed and imputed data, so that any patterns detected correspond to true qualitative features of the sequence–function relationship rather than artifacts due to the pattern of missing data. To evaluate the extent of the consistency between the in-sample and out-of-sample regions of sequence space, we first considered the phenomenon of model optimism^63^, where the in-sample *R*^2^ of a fit model can be far higher than its out-of-sample *R*^2^ (Fig. 4e). We see that while the *L*_2_ three-way and pairwise models have in-sample *R*^2^ that are roughly constant in sampling density and often far higher than the out-of-sample *R*^2^, our smoothed landscape provides a global fit wherein the in-sample and out-of-sample *R*^2^ are well-calibrated to each other, so that the goodness of fit is roughly constant across all of sequence space.

While Fig. 4e shows that the extent of model optimism for the pairwise and three-way interaction models is largely alleviated at high data densities, we paradoxically observed anti-conservative behavior for these models in the high data regime. In particular, when a large fraction of possible genotypes are used as training data, these models appear to suffer from an artifact wherein they have a tendency to predict local maxima at out-of-sample sequences, with this enrichment reaching greater than threefold when using our largest fraction of sequence space for training (92.4% of genotypes, Supplementary Fig. 4). In contrast, minimum epistasis interpolation does not exhibit this enrichment, and rather behaves conservatively, showing a depletion of out-of-sample predicted local maxima in the data-dense regime (Supplementary Fig. 4). Because in studies of sequence–function relationships we are often particularly interested in the positions of these local maxima (e.g. “fitness peaks”), the conservative behavior of minimum epistasis interpolation may be desirable in order to limit the number and frequency of false-positive predictions.

#### Structure of epistasis in protein G

We have shown that minimum epistasis interpolation combined with the smoother **M** has the tendency to remove experimental noise and spurious maxima while preserving the large-scale structure of the landscape and accommodating complex higher-order epistasis. This suggests that such methods may also be useful for the interpretation, exploration, and intuitive explanation of empirical data for specific sequence–function relationships. In this section, we combine imputation using minimum epistasis interpolation and the corresponding smoother **M** with a visualization technique developed in ref. ^47^ to perform exploratory data analysis on the full 20^4^ = 160,000 genotype GB1-binding landscape^34^. Problems with missing data and a proliferation of noise-driven local optima had previously impeded successful application of this visualization technique to the empirical data. We show that the methods used here alleviate these difficulties, allowing for a simple and intuitive analysis of this highly epistatic sequence–function mapping.

**Fig. 5:**
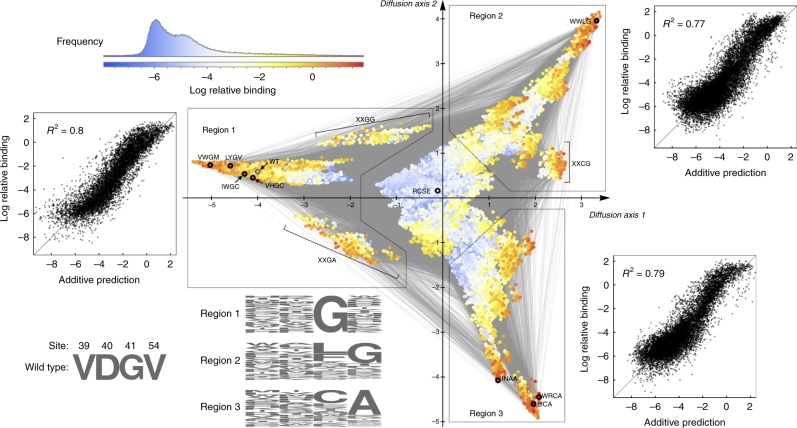
Visualization of the GB1 landscape reconstructed using minimum epistasis interpolation and the local nonepistatic smoother. Genotypes are plotted using the dimensionality reduction technique from ref. ^47^ (see “Methods”). Points are genotypes, colored according to their smoothed binding phenotype, and two genotypes are connected by an edge if they differ by a single amino acid substitution. Local fitness peaks are highlighted by black circles. The *x*- and *y*-axis are, respectively, the first and second diffusion coordinate and have units of square-root expected neutral substitutions per site. Three high-fitness regions are characterized by their distinct sequence composition (sequence logos, see Supplementary Fig. 5b for numerical values). The scatter plots show the fit of an additive model to the unsmoothed binding values within each of the three high-binding regions. These scatter plots indicate that, despite the complex pattern of epistasis in the landscape as a whole, the sequence–function relationship is approximately additive within each individual high-binding region.

In particular, the visualization technique is based on using the GB1 data to construct a model of molecular evolution for these four amino acid positions and creates a low-dimensional representation of the corresponding sequence space that optimally approximates the time for a population to evolve from one genotype to another under selection for high binding (see “Methods”). The result is a plot where high-binding (i.e. high-fitness) sequences are broadly separated when it would typically take a long time to evolve from one to the other. Figure 5 shows this visualization for GB1, and indicates that there are three relatively distinct sets of high-binding sequences (warm colors) that would take a long time to evolve from one to the other. These regions contain the vast majority of high-binding sequences (97.5% of sequences with smoothed fitnesses greater than wild type, and all of the top 100 measured binders are contained within the boxed regions) and appear as protrusions from a core of low-binding sequences (cool colors), plotted near the origin. The figure marks local maxima with black rings, and we see that each of these separate regions of high-binding sequences corresponds to a cluster of one or more local fitness maxima, with the wild-type sequence observed near one of these clusters (wild-type marked with gray ring).

To better understand the characteristics of these three high-binding regions and their underlying biophysical explanation, we constructed sequence logos to identify the common features of the sequences within each region (Fig. 5). We see that the key characteristic of the first region, which contains the wild-type sequence, is that they all have a glycine at position 41. However, the composition of the other two regions is more complex. Sequences in region 2 often have a glycine at position 54 instead of 41, and the highest binders tend to instead have a leucine or phenylalanine at site 41. In region 3, sequences typically have an alanine instead of a glycine at site 54, with the highest binders generally having either a cysteine of another alanine at site 41. At a more biophysical level, sites 41 and 54 directly interact in the GB1 crystal structure, albeit as part of dynamically active and conformationally variable portion of the protein^5,34^. In particular ref. ^5,34^, previously suggested that the epistasis observed at these sites was in part due to steric interactions between sites 41 and 54. This analysis is consistent with our observation that the major differences between the three high-binding regions lie in the identity (glycine or alanine) and placement (site 41 or 54) of the small non-polar residue relative to bulkier amino acids (e.g. leucine, phenylalanine, or cysteine at site 41).

Finally, we considered the structure of the fitness landscape within each of the high-binding regions. Perhaps surprisingly, we found that within each region even the unsmoothed values are reasonably approximated by a simple additive model (scatter plots in Fig. 5, *R*^2^ of 0.77–0.8, randomization test *p* < 0.003 for each region, see “Methods”). However, these models differ substantially between the three regions, particularly with respect to the additive effects of substitutions at sites 41 and 54 (Supplementary Fig. 5), and all these models fit substantially better than the global additive model investigated previously (*R*^2^ = 0.49), indicating that overall the sequence–function relationship appears to be locally rather than globally additive.

In summary, we are left with a qualitative understanding of the structure of this fitness landscape at several different levels of detail. At the coarsest level, we find that although the GB1 fitness landscape harbors a substantial degree of epistasis, in large part this arises from the presence of three distinct high-fitness regions, and that the fitness landscape is approximately additive within each such region. At a finer level of detail, we observe the presence of multiple local fitness maxima within some of these regions. Finally, our visualization (Fig. 5) provides a rich depiction of the finer-scale structure of the landscape, suggesting many hypotheses that are ripe for further exploration. For instance, the visualizations show what appear to be fitness “ridges” connecting one high-binding region to another (e.g. the XXGG sequences connecting Region 1 to Region 2, and the XXGA sequences connecting Region 1 to Region 3, respectively), that can serve as paths of moderate fitness that a population might be most likely to take when traversing from one high-fitness region to another. Importantly, these insights all depend on the application of our smoother, which simplifies the landscape by removing features attributable to experimental noise and fully random epistasis in order to reveal its large-scale features (see Supplementary Fig. 6 for an attempt at visualization using the incomplete, unsmoothed landscape).

### Application to transcription factor binding

**Fig. 6:**
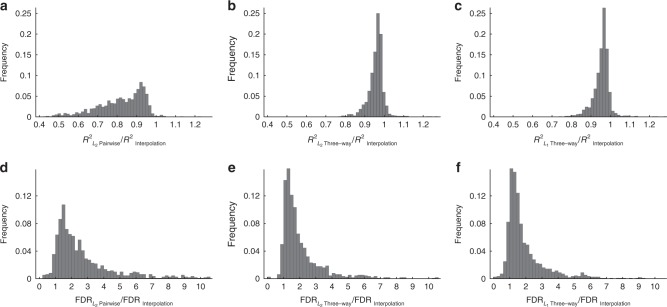
Model comparison using protein-binding microarray data from 1121 transcription factors^48^. For each TF, 80% of sequences were randomly assigned as training data. *L*_2_-regularized pairwise regression, *L*_2_-regularized three-way regression, and *L*_1_-regularized three-way regression were fit with regularization parameter chosen by cross-validation. For each TF, we calculate the out-of-sample *R*^2^ and false discovery rate (FDR) defined as the frequency that an out-of-sample genotype predicted to be above the 95th percentile of the data were in fact below the 95th percentile. **a**–**c** Histograms of the ratios of *R*^2^ of the regression models and minimum epistasis interpolation. **d**–**f** Histograms of the ratios of the false discovery rate of the regression models and minimum epistasis interpolation.

Finally, in order to observe the performance of the interpolation method across a wider variety of sequence–function relationships, we applied the interpolation method to protein-binding microarray data corresponding to the binding preferences of 1121 transcription factors^48^, a set of measurements that has previously served as a model system for studying the genotype–phenotype map^49,50^. In particular, these protein-binding microarray experiments produce a measure of the preference of the assayed transcription factor for all possible DNA sequences of length eight (4^8^ = 65,536 total sequences). The standard measure of this preference is known as the E-score^64,65^, which serves as a proxy for binding affinity. We downloaded all E-scores reported in ref. ^48^ from the Cis-BP database (http://cisbp.ccbr.utoronto.ca), and tested the ability of two-way and three-way *L*_2_-regularized regression, three-way *L*_1_-regularized regression, and interpolation models to predict a held-out set of E-scores when trained on 80% of the data. Figure 6a–c shows that the interpolation method has a superior out-of-sample *R*^2^ than these other models the vast majority of the time, and in fact had the best out-of-sample *R*^2^ of any of these models for 93.4% of transcription factors. Because we are especially interested in the accuracy of predictions for functional binding sites and previously observed that lower-order regression models sometimes make spurious, extreme out-of-sample predictions, we also considered the fraction of predictions of strong binding sequences (defined as predicted E-scores greater than the 95th-percentile of the data) that were in fact false-positive predictions. Figure 6d–f shows that the rate of these false positive predictions was often several fold lower for the interpolation method (median 2.2-fold and 1.5-fold reduction in false discovery rate compared with the *L*_2_ pairwise and *L*_2_ /*L*_1_ three-way models, respectively), which again confirms the conservative character of the minimum epistasis interpolation predictions.

## Discussion

Understanding the mapping from genotype to phenotype is a key problem for much of biology, from applied areas such as protein design^44,66^, antigenic evolution^67^, and the emergence of drug resistance^68^, to more basic questions about the repeatability of adaptation^69^ and the dynamics of long-term molecular evolution^31^. While the astronomical number of possible genotypes may put a fully comprehensive understanding of this mapping forever out of reach, modern high-throughput experiments are currently providing phenotypic measurements for tens of thousands to millions of genotypes at a time, so that there is a need for computational techniques to translate these high-throughput measurements into phenotypic predictions for genotypes that have not yet been assayed. Here, we have presented a principled and highly conservative solution to this problem by inferring the least epistatic possible sequence–function relationship compatible with the observed data in the sense that mutational effects change as little as possible between mutationally adjacent genetic backgrounds while exactly matching the data where available.

One simple way of understanding our approach is by contrasting it with the classical nonepistatic model^70^, since both models in some sense minimize the amount of epistasis, but do so in different ways. In a nonepistatic model, one assumes that the sequence–function relationship is completely additive so that the effects of mutations are constant across genetic backgrounds and, consequently, the mean-squared epistatic coefficient between random pairs of mutations across random backgrounds is constrained to be precisely zero. One then determines these mutational effects by minimizing the mean-squared error of the model predictions for genotypes where data is available.

In minimum epistasis interpolation, these choices are exactly reversed. Whereas a nonepistatic model minimizes the mean-square error under the constraint that the mean-square epistatic coefficient is precisely zero, here we constrain the reconstruction to exactly match the data so that the mean square error is precisely zero and infer the missing values by minimizing the mean-square epistatic coefficient. This allows the data itself to dictate the amount and character of epistasis that is included, since the reconstruction is as additive as possible while still being highly epistatic in regions of sequence space where the data require it. In the Supplementary Methods, we show that the classical nonepistatic model and minimum epistasis interpolation can actually be viewed as two ends of a continuum of models that minimize a convex combination of mean-square error and mean-square epistasis, and which all have out-of-sample properties similar to minimum epistasis interpolation. Furthermore, this generalized procedure allows one to tune the degree of regularization and to accommodate unequal noise variance among genotypes (see Supplementary Methods).

Our method also provides insights into the interpretation of higher-order epistatic interactions, that is interactions between mutations at three or more sites. When viewing genotype–phenotype mappings from a regression or analysis of variance standpoint, there is a tendency—going back to the very earliest days of statistics and experimental design^71,72^—to assume that higher-order interaction terms are likely to be small (e.g. in partial factorial designs where higher-order interactions are purposefully confounded with main effects and lower-order interactions^73^). However, there is a growing consensus that such higher-order interactions are not only common in genotype–phenotype maps^10,18,29,32,38^ but are expected even for very simple, smooth genotype–phenotype relationships, such as where the observed phenotype is just an additive trait that has been run through a nonlinear transformation^31,32,40,74–76^. Our results contribute to this view by showing that the incorporation of higher-order interactions in fact allows substantially less epistatic fits than standard pairwise models. To see why this is the case, it is helpful to realize that higher-order genetic interactions can be thought of as pairwise interactions whose strength changes over different regions of sequence space, which in particular allows the strength of pairwise epistatic interactions to decay toward zero in regions of sequence space that are data-poor or where the interaction is not supported.

Besides viewing genotype–phenotype maps as being defined by sums of interactions between sites as in regression models^26,29,77^, there are a rich variety of other formalisms for describing genetic interaction that are related to the techniques we have developed here^78–82^. Probably the most relevant of these is the correlation between the effects of mutations measured in mutationally adjacent genetic backgrounds, *γ*^10,81^. Conceptually, maximizing *γ* would be quite similar to our method except that *γ* depends on both $\overline{{\epsilon }^{2}}$ and the variance in the phenotypic effects of mutations^81^, so that maximizing *γ* would tend to inflate the magnitude of mutational effects, in essence minimizing the relative rather than absolute amount of epistasis. Our face-specific epistatic coefficients are also related to the “circuit” approach of ref. ^79^ in that these epistatic coefficients correspond to a subset of the possible circuits (specifically those corresponding to conditional epistasis). However, at a deeper level our approach is most closely related to the Walsh–Fourier decomposition^29,77,83–85^, where the phenotype is expanded in terms of the eigenvectors of the graph Laplacian **L**, which are also the eigenvectors of the second-order discrete Laplace operator **L**^2^ − *α***L**, so that our minimization problem can be re-cast as minimizing a weighted sum of squared Walsh coefficients, where the weight increases quadratically with interaction order (see Supplementary Methods). Finally, while boundary-value problems involving the graph Laplacian **L** arise in many areas of applied mathematics e.g.^86–88^, here we are faced with a more unusual boundary value problem for **L**^2^ − *α***L**. Interestingly, this second-order character arises because of our stipulation that the mutational effects—rather than the phenotypic values themselves—change smoothly as we move through sequence space. In contrast, naive interpolation based on **L** results in an unrealistic degree of sign epistasis where e.g., multiple deleterious mutations combine to be the average of the single mutations rather than their sum.

While our interpolation procedure exactly matches the data where available, some degree of smoothing is often helpful to better understand the large-scale features of the sequence–function relationship and to ameliorate the effects of experimental noise. To address this need, we proposed a smoother that is philosophically similar to LOESS^89^ in that it approximates the sequence–function relationship as being locally additive while making no assumptions about its global structure. Specifically, the smoother replaces the phenotypic value for each genotype with the average of the nonepistatic predictions that would be obtained by taking each possible double mutant as the wild type. Because of the large number of single and double mutants that this smoothed estimate averages over, such smoothing greatly decreases the impact of experimental noise. It is important to note that by the same argument the application of the smoother will largely remove any true fully random component of the sequence–function relationship (i.e. the so-called house-of-cards component^30,40,57^). Thus, for applications where we are most interested in genotypes with high phenotypic values (high fitness or highly functional genotypes), concordance between the smoothed and raw experimental phenotypes for a high functionality provides confidence not only that the genotype is likely to be truly functional, but also that this functionality is due to a consistent tendency in the local sequence–function relationship rather than some idiosyncratic feature of the individual genotype. Importantly, the out-of-sample predictions of our interpolation solution have similar noise-reduction properties to the smoother, since the out-of-sample predictions are invariant under the action of the smoother. To see the extent of this reduction, we note that the interpolation solution is linear in the data (see “Methods”, Eq. (9)) so that the influence of noise on the out-of-sample predictions can be assessed by conducting interpolation on a pure noise landscape. Conducting this analysis for the GB1 data set (4 sites, with 20 alleles per site), we find the variance due to noise in our out-of-sample predictions is reduced 20 to 50-fold relative to the noise in the original data (Supplementary Fig. 3).

Minimum epistasis interpolation provides a principled and highly conservative method for reconstructing sequence–function relationships that has no tunable parameters and allows epistatic interactions of all orders. Nonetheless, the method has a number of important limitations. First, precisely because of this conservative nature, the method requires a relatively high density of data to predict a substantial amount of epistasis. In the examples we have explored here, we see that in order to produce substantially epistatic predictions the method requires that at least some region of sequence space has a local sampling density greater than roughly 10%, and generally predicts negligible epistasis in regions of sequence space where less than 1% of genotypes have been observed (see also, Supplementary Fig. 8, which directly explores how the extent of epistasis depends on sampling density). Thus the method is best viewed as an interpolation or imputation procedure that extrapolates additively where data are sparse. Second, the method is somewhat more computationally intensive than lower-order regression models, and our present implementations are limited to sequence spaces containing less than roughly a million genotypes or, alternatively (using our kernelized implementation), studies containing less than roughly twenty thousand observed sequences. Third, we emphasize that despite its many interesting and useful properties, the method introduced here produces only the least epistatic possible reconstruction of the sequence–function relationship, and hence is almost necessarily underfitting the data. More general statistical approaches that better reflect the character of epistasis found in a specific data set are likely possible and capable of providing better out-of-sample performance.

## Methods

### Formulation of the minimization problem and its solution

Suppose our sequence space consists of two bi-allelic loci (*α* = 2, *l* = 2) and hence four possible genotypes {ab, aB, Ab, AB}. Given a vector that assigns phenotypes to all four genotypes, ${{\bf{f}}}^{T}={\left[{f}_{{\rm{ab}}}{f}_{{\rm{aB}}}{f}_{{\rm{Ab}}}{f}_{{\rm{AB}}}\right]}^{T}$, we can calculate the squared epistatic coefficient for **f** as

6 $${\epsilon }^{2}({\bf{f}})=	\, {(({f}_{{\rm{AB}}}-{f}_{{\rm{aB}}})-({f}_{{\rm{Ab}}}-{f}_{{\rm{ab}}}))}^{2}\\ =	\,{f}_{{\rm{AB}}}^{2}+{f}_{{\rm{aB}}}^{2}+{f}_{{\rm{Ab}}}^{2}+{f}_{{\rm{AB}}}^{2}\\ 	\! -2{f}_{{\rm{AB}}}{f}_{{\rm{aB}}}-2{f}_{{\rm{AB}}}{f}_{{\rm{Ab}}}-2{f}_{{\rm{aB}}}{f}_{{\rm{ab}}}-2{f}_{{\rm{Ab}}}{f}_{{\rm{ab}}}\\ 	\! +2{f}_{{\rm{AB}}}{f}_{{\rm{ab}}}+2{f}_{{\rm{aB}}}{f}_{{\rm{Ab}}}.$$

Extending this to arbitrary *α* and *l*, for a vector **f** defined on the set of all *α*^*l*^ genotypes we can calculate the mean-squared epistatic coefficient $\overline{{\epsilon }^{2}}({\bf{f}})$ by averaging over all $$s=\left(\begin{array}{l}l\\ 2\end{array}\right)\left(\begin{array}{l}\alpha\\ 2\end{array}\right)^{2}{\alpha }^{l-2}$$ faces of the sequence space. This results in a positive semi-definite quadratic form $\overline{{\epsilon }^{2}}({\bf{f}})=\frac{1}{s}{\sum }_{i,j}c(i,j){\bf{f}}(i){\bf{f}}(j)$, where the *c*(*i*, *j*) can be found by counting the number of times the ordered product **f**(*i*)**f**(*j*) appears when summing Eq.  (6) over all faces and which only depend on the Hamming distance *d*(*i*, *j*) between sequences *i* and *j*. First, the squared term for any given genotype (distance 0) appears in $$p=\left(\begin{array}{l}l\\ 2\end{array}\right){(\alpha -1)}^{2}$$faces with coefficient 1. Second, each ordered pair of genotypes that are at Hamming distance 1 from each other appear in (*l* − 1)(*α* − 1) faces with coefficient −1. Third, if the ordered pair of genotypes are separated by distance 2, they appear in exactly one face with coefficient 1. Thus, we arrive at Eq. (4) in the main text.

Now, arrange the coefficients *c*(*i*, *j*) in a matrix **C** with **C**(*i*, *j*) = *c*(*i*, *j*)/*s* and $\overline{{\epsilon }^{2}}({\bf{f}})={{\bf{f}}}^{T}{\bf{C}}{\bf{f}}$. Given data ${\bf{y}}\in {{\mathbb{R}}}^{m}$ for a subset of sequences *B* of size *m* of the set of all possible sequences ${\mathcal{S}}$, we write $I={\mathcal{S}}\setminus B$ to be the set of all missing sequences. Without loss of generality, we will order our sequences so that the *m* sequences in *B* whose phenotypes are known come first. Our aim is to infer a full landscape ${\widehat{{\bf{f}}}}^{T}=\left[{\widehat{{\bf{f}}}}_{B}^{T}\quad {\widehat{{\bf{f}}}}_{I}^{T}\right]$ that minimizes the average squared epistatic coefficient under the constraint that we do not change the values for genotypes in *B*. We can formulate this as a quadratic minimization problem with equality constraint:

7 $${\rm{minimize}}\;{{\bf{f}}}^{T}{\bf{C}}{\bf{f}},$$

8 $${\rm{subject}}\;\ {\rm{to}}\;{{\bf{f}}}_{B}={\bf{y}}.$$

Using *B* and *I* to index submatrices of **C**, we can solve this minimization problem by differentiating **f**^*T*^**Cf** with respect to **f**_*I*_ and setting the gradient to zero. This gives us:

9 $$\widehat{{\bf{f}}}=\left[\begin{array}{l}{\widehat{{\bf{f}}}}_{B}\\ {\widehat{{\bf{f}}}}_{I}\end{array}\right]=\left[\begin{array}{l}{\bf{y}}\\ -{({{\bf{C}}}_{II})}^{-1}{{\bf{C}}}_{IB}{\bf{y}}\end{array}\right],$$

which is equivalent to Eq. (3) in the main text (the matrix ${({{\bf{C}}}_{II})}^{-1}$ exists if and only if the nonepistatic model fit by least squares has a unique solution, see Supplementary Methods Proposition 1). Note that by Eq. (9), the predictions ${\widehat{{\bf{f}}}}_{I}$ are simply a linear transformation of the observations *y*. Thus, if the observations *y* contain i.i.d. noise with mean 0 and variance *σ*^2^ then Eq. (9) gives the mean of ${\widehat{{\bf{f}}}}_{I}$ while the covariance matrix is given by

10 $${\rm{cov}}({\widehat{{\bf{f}}}}_{I})={\sigma }^{2}{({{\bf{C}}}_{II})}^{-1}{{\bf{C}}}_{IB}{{\bf{C}}}_{BI}{({{\bf{C}}}_{II})}^{-1},$$

a matrix whose main diagonal gives the noise-induced variance in the individual out-of-sample predictions.

### Mathematical properties of the solution

Rearranging Eq. (9) as ${{\bf{C}}}_{II}{\widehat{{\bf{f}}}}_{I}-{{\bf{C}}}_{IB}{\widehat{{\bf{f}}}}_{B}=0$, we find the solution $\widehat{{\bf{f}}}$ must satisfy $({\bf{C}}\widehat{{\bf{f}}})(i)=0$, for all genotypes *i* in *I*. To understand what this condition means, we rescale our cost matrix and use it to define a new matrix  ${\bf{M}} = {\bf{I}} - \frac{s}{p}{\bf{C}}$, where **I** is the identity matrix. Using the definition of **C** and Eq. (4) gives us

11 $$({\bf{M}}{\bf{f}})(i)=\frac{1}{p}\left(\sum_{j:d(i,j) = 1}(l-1)(\alpha -1){\bf{f}}(j)-\sum_{k:d(i,k) = 2}{\bf{f}}(k)\right)$$

12 $$=\frac{1}{p}\sum_{F:i\in F}\sum_{j,k,l\in F\setminus \{i\}}{\bf{f}}(j)+{\bf{f}}(k)-{\bf{f}}(l),$$

where we enumerate through all *p* faces *F* that *i* belongs to. For each face *F*, **f**(*j*) + **f**(*k*) − **f**(*l*) = **f**(*j*) − **f**(*l*) + **f**(*k*) − **f**(*l*) + **f**(*l*) is the nonepistatic prediction based on sequences *j*, *k* which are at distance 1 to *i* and *l* which is at distance 2 to *i* (Eq. (2)). Therefore, (**Mf**) (*i*) returns the average local nonepistatic prediction for *i* based on all faces containing *i*. Thus the necessary condition $({\bf{C}}\widehat{{\bf{f}}})(i)=0$ is equivalent to $\widehat{{\bf{f}}}(i)=({\bf{M}}\widehat{{\bf{f}}})(i)$,  ∀*i* ∈ *I*. That is, for any unknown genotype *i*, its inferred value must be equal to its average local nonepistatic prediction based on $\widehat{{\bf{f}}}$. As a result, applying **M** to $\widehat{{\bf{f}}}$ does not alter our predictions for the out-of-sample genotypes.

Let **d**_*k*_(*i*) denote the mean value of $\widehat{{\bf{f}}}$ for sequences at distance *k* to *i*. We can rewrite Eq. (11) as

13 $$({\bf{M}}\widehat{{\bf{f}}})(i)= \, \frac{1}{p}(l-1)(\alpha -1)\sum_{j:d(i,j) = 1}\widehat{{\bf{f}}}(j)-\frac{1}{p}\sum_{k:d(i,j) = 2}\widehat{{\bf{f}}}(k)$$

14 $$\,\,\, = \,\frac{1}{p}(l-1)(\alpha -1)l(\alpha -1){{\bf{d}}}_{1}(i)-{{\bf{d}}}_{2}(i)$$

15 $$= \, {{\bf{d}}}_{1}(i)+({{\bf{d}}}_{1}(i)-{{\bf{d}}}_{2}(i)),$$

which gives us a geometric interpretation for our method (Eq. (5) in the main text).

Our minimization problem also has a close relation to the Dirichlet problem on a graph^88^. To see this, recall the definition of the graph Laplacian **L** for our Hamming graph of all possible sequences

16 $${\bf{L}}(i,j)=\left\{\begin{array}{ll}l(\alpha -1)&i=j\\ -1&d(i,j)=1\\ 0&{\rm{otherwise}}.\end{array}\right.$$

A discrete Dirichlet problem is formulated as finding a function $\widehat{{\bf{f}}}$ defined on the graph so that $({\bf{L}}\widehat{{\bf{f}}})(i)=0$ on the unsampled genotypes (interior) *i* ∈ *I*, while satisfying the condition ${\widehat{{\bf{f}}}}_{B}={\bf{y}}$.

It turns out that we can re-express **C** in terms of **L** and **L**^2^. In particular for **L**^2^, we have

17 $${{\bf{L}}}^{2}(i,j)=\left\{\begin{array}{ll}{(l(\alpha -1))}^{2}+l(\alpha -1)&i=j\\ -2l(\alpha -1)+(\alpha -2)&d(i,j)=1\\ 2&d(i,j)=2\\ 0&{\rm{otherwise}}.\end{array}\right.$$

Consequently, **L**^2^ − *α***L** = 2*s***C**. Thus, instead of being harmonic in the interior, i.e. $({\bf{L}}\widehat{{\bf{f}}})(i)=0$, as in the classical discrete Dirichlet problem, the solution to our problem instead must satisfy $(({{\bf{L}}}^{2}-\alpha {\bf{L}})(\widehat{{\bf{f}}}))(i)=0$ for *i* ∈ *I*, in addition to the boundary-value constraint.

### Simulation of crater landscape

We simulated data under the crater landscape model^46^ for the fitness of a transcription factor binding site. Specifically, we assume the effects of mutations on binding energy to be constant and the binding probability of any sequence is a function of its Hamming distance *d* to the best binding sequence, $p=\frac{1}{1+{e}^{\epsilon (d-\rho )}}$, where *ϵ* is the binding energy per nucleotide mismatch and the compound parameter *ϵρ* is the chemical potential measuring the factor concentration^46^. In this minimal model, we assume there are two cellular states. The on state favors the expression of the gene, and hence selects for high-binding probability with selection coefficient *s*_on_. The off state disfavors gene expression and selects against high binding with coefficient *s*_off_ = −*s*_on_. The total fitness of a sequence at distance *d* is given by

18 $$f(d)=\frac{{s}_{{\rm{on}}}}{1+{e}^{\epsilon (d-{\rho }_{{\rm{on}}})}}-\frac{{s}_{{\rm{on}}}}{1+{e}^{\epsilon (d-{\rho }_{{\rm{off}}})}}.$$

We choose the following parameters *ϵ* = 1, *ρ*_on_ = 6, *ρ*_off_ = 1, and *s*_on_ = 1. We use this model for simulating fitness landscape data for the set of all possible mutants corresponding to a sequence space with *l* = 16 sites and two allelic states at each site.

### Sparse interaction model

We first simulated sparse interaction landscapes using the formula

19 $${\bf{f}}={\bf{X}}{\boldsymbol{\theta }},$$

where **X** is the one-hot design matrix with columns corresponding to specific allelic combinations in specific subsets of sites. For any given column, the value of a genotype is 1 if it contains the corresponding allelic combination on the prescribed sites, and 0 otherwise. We consider genetic interactions of all orders, so that the dimension of *X* is *α*^*l*^ × (*α* + 1)^*l*^, since $$\mathop{\sum }\nolimits_{k = 0}^{l}\left(\begin{array}{l}l\\ k\end{array}\right){\alpha }^{k}={(\alpha +1)}^{l}$$. ***θ*** is the (*α* + 1)^*l*^-dimensional vector of coefficients. To ensure sparsity, we randomly set 90% of the entries of ***θ*** to 0, and sampled the remaining 10% independently from the standard normal distribution.

### *L*_2_-regularized regression

We use the following linear model to fit pairwise and three-way interaction models to the GB1 data set.

20 $$\widehat{{\bf{f}}}(i)=\mathop{\sum }_{j}{\phi }_{ij}{\beta }_{j}+\mathop{\sum }_{k}{\psi }_{ik}{\gamma }_{k},$$

or in matrix notation

21 $$\widehat{{\bf{f}}}={\boldsymbol{\Phi }}{\boldsymbol{\beta }}+{\boldsymbol{\Psi }}{\boldsymbol{\gamma }}.$$

The matrix Φ has as columns any orthonormal set of vectors that span the space of nonepistatic fitness landscapes (eigenspace of the graph Laplacian **L** associated with eigenvalues 0 and *α*). The columns of **Ψ** form an orthonormal set of vectors that span the space of all pairwise or pairwise and three-way functions (eigenspace of **L** associated with eigenvalue 2*α* (pairwise) or 2*α* and 3*α* (three-way), see Supplementary Methods).

We fitted *L*_2_-regularized pairwise and three-way regression models to different training data sets *B*. Specifically, we find our solution by minimizing

22 $$\sum_{i\in B}{\left({y}_{i}-\left(\sum_{j}{\phi }_{ij}{\beta }_{j}+\sum_{k}{\psi }_{ik}{\gamma }_{k}\right)\right)}^{2}+\lambda | | {\boldsymbol{\gamma }}| {| }^{2}.$$

The regularization parameter *λ* is chosen from a set of potential parameters equally spaced on the ${\mathrm{log}\,}_{10}$ scale. For each training sample, we performed 10-fold cross-validation to generate average mean squared errors using the candidate *λ*’s. The *λ* with the lowest cross-validated MSE is used to fit the training data and make predictions for the test data set.

### *L*_1_-regularized regression

In addition to the *L*_2_ regularized regressions, we also fit *L*_1_ regularized three-way regression models, where the design matrix is given by the columns of the one-hot matrix **X** corresponding to interactions between up to three sites. The models were fit using penalized least squares with the penalty given by the *L*_1_ norm of the coefficients. We used the R package glmnet^90^ to fit *L*_1_ regularized regression models for all data sets with regularization parameter chosen by cross-validation using the default setting.

### Visualization of the GB1 landscape

We consider a population evolving in continuous time under weak mutation e.g., refs. ^91–93^ on the full 20^4^ = 160,000 genotype GB1-binding landscape smoothed using **M**. Specifically, we model evolution as a continuous-time Markov chain where the population moves from genotype to genotype at each fixation event. The rate matrix *Q* of the Markov chain is

23 $$Q(i,j)=\left\{\begin{array}{ll}\frac{1}{\alpha -1}\frac{c({\bf{f}}(j)-{\bf{f}}(i))}{1-{e}^{-c({\bf{f}}(j)-{\bf{f}}(i))}}&d(i,j)=1\\ -\mathop{\sum}\nolimits_{k\ne i}Q(i,k)&i=j\\ 0&{\rm{otherwise}},\end{array}\right.$$

where *c* is the conversion factor that transforms log binding to scaled fitness (Malthusian fitness × *N*_*e*_). We choose *c* so that the expected log binding at stationarity is equal to the log binding of the wild type. Time is scaled so that the total mutation rate per site is equal to 1. For a two-dimensional representation of the GB1 landscape, we use as coordinates the right eigenvectors of *Q* associated with the two largest nonzero eigenvalues. This allows our low-dimensional representation of the landscape to optimally capture the expected time for a population to evolve from genotype *i* to *j*^47^.

### Significance test of *R*^2^ for local additive fits

To assess the statistical significance of the total *R*^2^’s of additive models fit to the three regions identified in our visualization of the GB1 landscape, we sampled, for each region, 1000 random subsets of the same size. We then fit additive models to these random subsets to calculate the null distribution of total *R*^2^. We calculate the *p*-value for the *R*^2^ for each region as the fraction of random subsets that have equal to or greater *R*^2^ than the observed value.

### Reporting summary

Further information on research design is available in the Nature Research Reporting Summary linked to this article.

## Supplementary information

Supplementary information

Supplementary Software 1

Description of Additional Supplementary Files

Reporting Summary

## Data Availability

The GB1 data can be downloaded at 10.7554/eLife.16965.024. E-scores of the protein binding microarray data can be accessed via the Cis-BP database (http://cisbp.ccbr.utoronto.ca, Database Build 0.90).

## Code availability

The Mathematica notebook and R files used in this study are included as Supplementary Software 1. The scripts will reproduce all figures in the article.

## References

[1] Kinney JB, McCandlish DM (2019). Massively parallel assays and quantitative sequence-function relationships. Annu. Rev. Genomics. Hum. Genet..

[2] Fowler DM (2010). High-resolution mapping of protein sequence-function relationships. Nat. Methods.

[3] Starita LM (2013). Activity-enhancing mutations in an E3 ubiquitin ligase identified by high-throughput mutagenesis. Proc. Natl Acad. Sci. USA.

[4] Melamed D, Young DL, Gamble CE, Miller CR, Fields S (2013). Deep mutational scanning of an RRM domain of the Saccharomyces cerevisiae poly (A)-binding protein. RNA.

[5] Olson CA, Wu NC, Sun R (2014). A comprehensive biophysical description of pairwise epistasis throughout an entire protein domain. Curr. Biol..

[6] Doud MB, Ashenberg O, Bloom JD (2015). Site-specific amino acid preferences are mostly conserved in two closely related protein homologs. Mol. Biol. Evol..

[7] Podgornaia AI, Laub MT (2015). Pervasive degeneracy and epistasis in a protein-protein interface. Science.

[8] Sarkisyan KS (2016). Local fitness landscape of the green fluorescent protein. Nature.

[9] Steinberg B, Ostermeier M (2016). Shifting fitness and epistatic landscapes reflect trade-offs along an evolutionary pathway. J. Mol. Biol..

[10] Bank C, Matuszewski S, Hietpas RT, Jensen JD (2016). On the (un)predictability of a large intragenic fitness landscape. Proc. Natl Acad. Sci. USA.

[11] Starr TN, Picton LK, Thornton JW (2017). Alternative evolutionary histories in the sequence space of an ancient protein. Nature.

[12] Pokusaeva VO (2019). An experimental assay of the interactions of amino acids from orthologous sequences shaping a complex fitness landscape. PLoS Genet..

[13] Plesa C, Sidore AM, Lubock NB, Zhang D, Kosuri S (2018). Multiplexed gene synthesis in emulsions for exploring protein functional landscapes. Science.

[14] Pitt JN, Ferré-D’Amaré AR (2010). Rapid construction of empirical rna fitness landscapes. Science.

[15] Jiménez JI, Xulvi-Brunet R, Campbell GW, Turk-MacLeod R, Chen IA (2013). Comprehensive experimental fitness landscape and evolutionary network for small RNA. Proc. Natl Acad. Sci. USA.

[16] Puchta O (2016). Network of epistatic interactions within a yeast snoRNA. Science.

[17] Li C, Qian W, Maclean CJ, Zhang J (2016). The fitness landscape of a tRNA gene. Science.

[18] Domingo J, Diss G, Lehner B (2018). Pairwise and higher-order genetic interactions during the evolution of a tRNA. Nature.

[19] Kinney JB, Murugan A, Callan CG, Cox EC (2010). Using deep sequencing to characterize the biophysical mechanism of a transcriptional regulatory sequence. Proc. Natl Acad. Sci. USA.

[20] Rosenberg AB, Patwardhan RP, Shendure J, Seelig G (2015). Learning the sequence determinants of alternative splicing from millions of random sequences. Cell.

[21] Julien P, Miñana B, Baeza-Centurion P, Valcárcel J, Lehner B (2016). The complete local genotype-phenotype landscape for the alternative splicing of a human exon. Nat. Commun..

[22] Ke S (2018). Saturation mutagenesis reveals manifold determinants of exon definition. Genome Res..

[23] Phillips PC (2008). Epistasis–the essential role of gene interactions in the structure and evolution of genetic systems. Nat. Rev. Genet..

[24] Kondrashov DA, Kondrashov FA (2015). Topological features of rugged fitness landscapes in sequence space. Trends Genet..

[25] Domingo J, Baeza-Centurion P, Lehner B (2019). The causes and consequences of genetic interactions (epistasis). Annu. Rev. Genomics Hum. Genet..

[26] Hinkley T (2011). A systems analysis of mutational effects in HIV-1 protease and reverse transcriptase. Nat. Genet..

[27] Kouyos RD (2011). Assessing predicted HIV-1 replicative capacity in a clinical setting. PLoS Pathog..

[28] Mostowy R (2012). Estimating the fitness cost of escape from HLA presentation in HIV-1 protease and reverse transcriptase. PLoS Comput. Biol..

[29] Weinreich DM, Lan Y, Wylie CS, Heckendorn RB (2013). Should evolutionary geneticists worry about higher-order epistasis. Curr. Opin. Genet. Dev..

[30] Neidhart J, Szendro IG, Krug J (2013). Exact results for amplitude spectra of fitness landscapes. J. Theor. Biol..

[31] Starr TN, Thornton JW (2016). Epistasis in protein evolution. Protein Sci..

[32] Sailer ZR, Harms MJ (2017). Detecting high-order epistasis in nonlinear genotype-phenotype maps. Genetics.

[33] Sailer ZR, Harms MJ (2017). High-order epistasis shapes evolutionary trajectories. PLoS Comput. Biol..

[34] Wu N, Dai L, Olson CA, Lloyd-Smith JO, Sun R (2016). Adaptation in protein fitness landscapes is facilitated by indirect paths. eLife.

[35] Echave J, Wilke CO (2017). Biophysical models of protein evolution: understanding the patterns of evolutionary sequence divergence. Annu. Rev. Biophys..

[36] Poelwijk FJ, Socolich M, Ranganathan R (2019). Learning the pattern of epistasis linking genotype and phenotype in a protein. Nat. Commun..

[37] Canale AS, Cote-Hammarlof PA, Flynn JM, Bolon DNA (2018). Evolutionary mechanisms studied through protein fitness landscapes. Curr. Opin. Struct. Biol..

[38] Weinreich DM, Lan Y, Jaffe J, Heckendorn RB (2018). The influence of higher-order epistasis on biological fitness landscape topography. J. Stat. Phys..

[39] Storz JF (2018). Compensatory mutations and epistasis for protein function. Curr. Opin. Struct. Biol..

[40] Otwinowski J, McCandlish DM, Plotkin JB (2018). Inferring the shape of global epistasis. Proc. Natl Acad. Sci. USA.

[41] Kryazhimskiy S, Rice DP, Jerison ER, Desai MM (2014). Global epistasis makes adaptation predictable despite sequence-level stochasticity. Science.

[42] Bedbrook CN, Yang KK, Rice AJ, Gradinaru V, Arnold FH (2017). Machine learning to design integral membrane channelrhodopsins for efficient eukaryotic expression and plasma membrane localization. PLoS Comput. Biol..

[43] Biswas, S. et al. Toward machine-guided design of proteins. Preprint at 10.1101/337154v1 (2018).

[44] Yang KK, Wu Z, Arnold FH (2019). Machine-learning-guided directed evolution for protein engineering. Nat. Methods.

[45] Romero PA, Krause A, Arnold FH (2013). Navigating the protein fitness landscape with Gaussian processes. Proc. Natl Acad. Sci. USA.

[46] Berg J, Willmann S, Lässig M (2004). Adaptive evolution of transcription factor binding sites. BMC Evol. Biol..

[47] McCandlish DM (2011). Visualizing fitness landscapes. Evolution.

[48] Weirauch MT (2014). Determination and inference of eukaryotic transcription factor sequence specificity. Cell.

[49] Payne JL, Wagner A (2014). The robustness and evolvability of transcription factor binding sites. Science.

[50] Aguilar-Rodríguez J, Payne JL, Wagner A (2017). A thousand empirical adaptive landscapes and their navigability. Nat. Ecol. Evol..

[51] Shah P, McCandlish DM, Plotkin JB (2015). Contingency and entrenchment in protein evolution under purifying selection. Proc. Natl Acad. Sci. USA.

[52] Boyd, S. & Vandenberghe, L. *Convex Optimization* (Cambridge University Press, 2004).

[53] Bookstein FL (1989). Principal warps: thin-plate splines and the decomposition of deformations. IEEE Trans. Pattern Anal. Mach. Intell..

[54] Mitteroecker P, Gunz P (2009). Advances in geometric morphometrics. Evol. Biol..

[55] Blows MW, Brooks R, Kraft PG (2003). Exploring complex fitness surfaces: multiple ornamentation and polymorphism in male guppies. Evolution.

[56] Martin CH, Wainwright PC (2013). Multiple fitness peaks on the adaptive landscape drive adaptive radiation in the wild. Science.

[57] Kingman J (1978). A simple model for the balance between selection and mutation. J. Appl. Probab. Stat..

[58] Mustonen V, Kinney JB, Callan CG, Lässig M (2008). Energy-dependent fitness: a quantitative model for the evolution of yeast transcription factor binding sites. Proc. Natl Acad. Sci. USA.

[59] Tenaillon O (2014). The utility of Fisher’s geometric model in evolutionary genetics. Annu. Rev. Ecol. Evol. Syst..

[60] Otwinowski J, Nemenman I (2013). Genotype to phenotype mapping and the fitness landscape of the *E. coli* lac promoter. PLoS ONE.

[61] Otwinowski J (2018). Biophysical inference of epistasis and the effects of mutations on protein stability and function. Mol. Biol. Evol..

[62] Nisthal A, Wang CY, Ary ML, Mayo SL (2019). Protein stability engineering insights revealed by domain-wide comprehensive mutagenesis. Proc. Natl Acad. Sci. USA.

[63] Efron B (1986). How biased is the apparent error rate of a prediction rule. J. Am. Stat. Assoc..

[64] Berger MF (2006). Compact, universal DNA microarrays to comprehensively determine transcription-factor binding site specificities. Nat. Biotechnol..

[65] Badis G (2009). Diversity and complexity in DNA recognition by transcription factors. Science.

[66] Badenhorst CP, Bornscheuer UT (2018). Getting momentum: from biocatalysis to advanced synthetic biology. Trends Biochem. Sci..

[67] Lässig M, Mustonen V, Walczak AM (2017). Predicting evolution. Nat. Ecol. Evol..

[68] Weinreich DM, Delaney NF, DePristo MA, Hartl DL (2006). Darwinian evolution can follow only very few mutational paths to fitter proteins. Science.

[69] De Visser JAG, Krug J (2014). Empirical fitness landscapes and the predictability of evolution. Nat. Rev. Genet..

[70] Fisher RA (1918). The correlation between relatives on the supposition of mendelian inheritance. Trans. R. Soc. Edinb..

[71] Fisher, R. A. *The Design of Experiments* (Oliver And Boyd, Edinburgh, 1935).

[72] Yates F (1937). The Design and Analysis of Factorial Experiments.

[73] Finney DJ (1943). The fractional replication of factorial arrangements. Ann. Eugen..

[74] Kondrashov FA, Kondrashov AS (2001). Multidimensional epistasis and the disadvantage of sex. Proc. Natl Acad. Sci. USA.

[75] Hartl DL (2014). What can we learn from fitness landscapes?. Curr. Opin. Microbiol..

[76] Diss G, Lehner B (2018). The genetic landscape of a physical interaction. eLife.

[77] Poelwijk FJ, Krishna V, Ranganathan R (2016). The context-dependence of mutations: a linkage of formalisms. PLoS Comput. Biol..

[78] Weinreich DM, Watson RA, Chao L (2005). Perspective: sign epistasis and genetic costraint on evolutionary trajectories. Evolution.

[79] Beerenwinkel N, Pachter L, Sturmfels B (2007). Epistasis and shapes of fitness landscapes. Stat. Sin..

[80] Szendro IG, Schenk MF, Franke J, Krug J, de Visser JAG (2013). Quantitative analyses of empirical fitness landscapes. Theory Exp..

[81] Ferretti L (2016). Measuring epistasis in fitness landscapes: The correlation of fitness effects of mutations. J. Theor. Biol..

[82] Ferretti L, Weinreich D, Tajima F, Achaz G (2018). Evolutionary constraints in fitness landscapes. Heredity.

[83] Stadler PF, Happel R (1999). Random field models for fitness landscapes. J. Math. Biol..

[84] Stadler, P. F. Fitness landscapes. in *Biological Evolution and Statistical Physics. *(eds Lässig, M. & Valleriani, A.) 183–204 (Springer-Verlag, 2002).

[85] Weinberger ED (1991). Fourier and Taylor series on fitness landscapes. Biol. Cybern..

[86] Bertalmio, M., Sapiro, G., Caselles, V. & Ballester, C. Image inpainting. in* Proceedings of the 27th Annual Conference on Computer Graphics and Interactive Techniques*. (eds Brown, J. R. & Akeley, K.) 417–424 (ACM Press/Addison-Wesley Publishing Co., 2000).

[87] Grady L (2006). Random walks for image segmentation. IEEE Trans. Pattern Anal. Mach. Intell..

[88] Biggs N (1997). Algebraic potential theory on graphs. Bull. Lond. Math. Soc..

[89] Cleveland WS (1979). Robust locally weighted regression and smoothing scatterplots. J. Am. Stat. Assoc..

[90] Friedman J, Hastie T, Tibshirani R (2010). Regularization paths for generalized linear models via coordinate descent. J. Stat. Softw..

[91] Iwasa Y (1988). Free fitness that always increases in evolution. J. Theor. Biol..

[92] Sella G, Hirsh AE (2005). The application of statistical physics to evolutionary biology. Proc. Natl Acad. Sci. USA.

[93] McCandlish DM, Shah P, Plotkin JB (2016). Epistasis and the dynamics of reversion in molecular evolution. Genetics.

