# Supplementary material for: Minimum epistasis interpolation for sequence-function relationships
Source: Nat Commun. 2020 Apr 14;11:1782. doi: 10.1038/s41467-020-15512-5 (PMC7156698; doi:10.1038/s41467-020-15512-5)
Supplement: Supplementary file 3 — Description of Additional Supplementary Files [file 41467_2020_15512_MOESM3_ESM.pdf]

Title: Supplementary Software 1
Description: Computational scripts to reproduce all data analyses and figures.
